# Roles of the Amino Terminal Region and Repeat Region of the *Plasmodium berghei* Circumsporozoite Protein in Parasite Infectivity

**DOI:** 10.1371/journal.pone.0032524

**Published:** 2012-02-29

**Authors:** Cassandra Aldrich, Alessandro Magini, Carla Emiliani, Tania Dottorini, Francesco Bistoni, Andrea Crisanti, Roberta Spaccapelo

**Affiliations:** 1 Department of Experimental Medicine, University of Perugia, Perugia Italy; 2 Department of Life Sciences, Imperial College London, London, United Kingdom; 3 Keble College, University of Oxford, Oxford, United Kingdom; Bernhard Nocht Institute for Tropical Medicine, Germany

## Abstract

The circumsporozoite protein (CSP) plays a key role in malaria sporozoite infection of both mosquito salivary glands and the vertebrate host. The conserved Regions I and II have been well studied but little is known about the immunogenic central repeat region and the N-terminal region of the protein. Rodent malaria *Plasmodium berghei* parasites, in which the endogenous CS gene has been replaced with the avian *Plasmodium gallinaceum* CS (*PgCS*) sequence, develop normally in the *A. stephensi* mosquito midgut but the sporozoites are not infectious. We therefore generated *P. berghei* transgenic parasites carrying the *PgCS* gene, in which the repeat region was replaced with the homologous region of *P. berghei* CS (*PbCS*). A further line, in which both the N-terminal region and repeat region were replaced with the homologous regions of *PbCS*, was also generated. Introduction of the *PbCS* repeat region alone, into the *PgCS* gene, did not rescue sporozoite species-specific infectivity. However, the introduction of both the *PbCS* repeat region and the N-terminal region into the *PgCS* gene completely rescued infectivity, in both the mosquito vector and the mammalian host. Immunofluorescence experiments and western blot analysis revealed correct localization and proteolytic processing of CSP in the chimeric parasites. The results demonstrate, *in vivo,* that the repeat region of *P. berghei* CSP, alone, is unable to mediate sporozoite infectivity in either the mosquito or the mammalian host, but suggest an important role for the N-terminal region in sporozoite host cell invasion.

## Introduction

The circumsporozoite protein (CSP) is the predominant surface antigen of *Plasmodium* sporozoites and is highly immunogenic being one of the key targets recognised by the host immune system. Pre-erythrocytic malaria vaccines have been primarily based on CSP [1], [2]. In particular, the central repeat region of *P. falciparum* CSP, which contains an immunodominant B cell epitope, represented the target of the first two vaccine trials [3], [4]. Recently, the results of a phase III trial of the RTS,S vaccine, based on both the repeat region and T-cell epitopes from the C-terminal region, provided evidence for protection against both clinical and severe malaria in African children [5].

CSP performs numerous functions for the sporozoite at different stages in the life cycle. The protein is first detected at high levels in the oocyst and has been shown to be vital in the process of sporogenesis [6], [7]. CSP is likely to be important in sporozoite gliding motility although the precise role that the protein plays in gliding remains unknown. Antibodies against CSP inhibit gliding motility [8] and sporozoites leave behind trails of CSP that correspond to their pattern of movement [9]. CSP has long been known to be involved in sporozoite infectivity [10], [11]. Specifically, it appears to be important in the binding of the sporozoite to both mosquito salivary glands [12], [13], [14] and vertebrate host hepatocytes [15], [16], [17].

Comparison of the deduced amino acid sequences of CS proteins from all species of *Plasmodium* shows that they have a similar overall structure. They all contain a central repeat region, whose amino acid sequence is species specific, and two conserved regions: a five amino acid sequence called Region I (RI), immediately upstream of the repeats, and a known cell-adhesive sequence with similarity to the type I repeat of thrombospondin called Region II (RII), downstream of the repeat region. CSP has a canonical glycosylphosphatidylinositol (GPI) anchor addition sequence in its C-terminus. Much evidence has been gathered on the functions of the conserved Regions I and II of CSP, as well as on residues outside of RI and RII, within the N- and C-terminal portions of the protein, which have been implicated in host binding [18], [19], [20], [21]. However, little evidence exists to date on the role of the CSP repeat region in the parasite life cycle.

**Figure 1:**
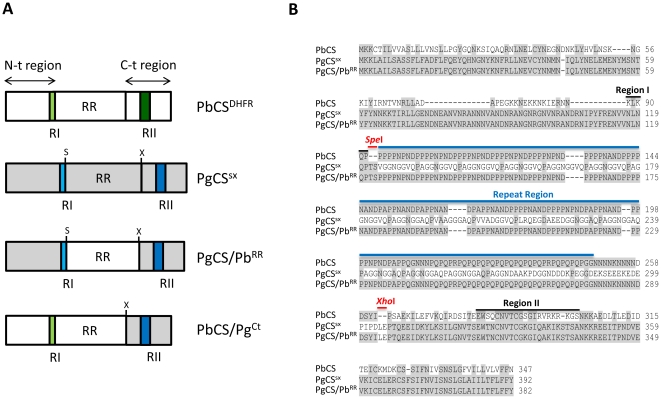
Schematic representation and alignment of wild type and transgenic *P.berghei* circumsporozoite proteins. (A) Schematic representation of the CS proteins present in each of the four transgenic *P. berghei* parasite lines generated. The corresponding names of the transgenic parasite lines are shown on the right. PbCS^DHFR^ parasites carry the wildtype PbCS coding sequence (white boxes, RI and RII indicated with light and dark green respectively) and, similar to all transgenic lines, contain the *T. gondii* DHFR drug selectable marker inserted in the CS locus. PgCS^SX^ parasites carry the full PgCS coding sequence (grey boxes, RI and RII indicated with light and dark blue respectively), but with the *Spe*I (S) and *Xho*I (X) restriction endonuclease sites inserted on either side of the repeat region. PgCS/Pb^RR^ parasites contain the PgCS N-terminal and C-terminal regions (grey boxes) and the PbCS repeat region (white box). PbCS/Pg^CT^ parasites carry the PbCS N-terminal and repeat regions (white boxes) and the PgCS C-terminal region (grey box). (B) Alignment of the wild type PbCS and transgenic PgCS^SX^ and PgCS/Pb^RR^ amino acid sequences. Shaded boxes represent areas of amino acid identity. Regions I and II (black lines), the repeat region (blue line) and the *Spe*I and *Xho*I restriction sites (red lines) are labelled. The *Spe*I and *Xho*I sites were introduced into the PgCS sequence to mediate exchange of the PgCS repeat region with the PbCS repeat region.

The RI core is a five amino acid sequence (KLKQP), conserved in almost all *Plasmodium* species with the exception of the avian malaria parasite *P. gallinaceum,* in which there are two amino acid changes in the core (NLNQP) (Figure 1). RI has been strongly implicated in the binding of salivary glands [13], [22]. A peptide encompassing RI was shown to inhibit binding of both recombinant CSP and sporozoites to the salivary glands. However, the RI core alone could not achieve this inhibition and upstream residues were also required [13], [22]. The sequence upstream of RI contains stretches of positively charged residues which are implicated, together with RI, in the interaction with liver heparan sulphate proteoglycans (HSPGs) [19], [21]. RI and RII peptides inhibited *P. falciparum* CSP (PfCSP) binding to HepG2 cell HSPGs to a similar extent, suggesting they both contribute to the interaction [23]. RII is a common motif present in the proteins of a wide range of organisms and is also found in another sporozoite surface protein, the thrombospondin-related adhesive protein (TRAP). It is an 18 amino acid region and part of a larger type I repeat of human thrombospondin (TSR) domain which acts as an adhesive module and binds with high affinity to heparin and certain sulphated glycoconjugates (Figure 1). The rapid and specific homing to the liver by sporozoites of mammalian *Plasmodium* spp. has been ascribed to the interaction between RII and glycosaminoglycan (GAG) side chains on HSPGs, located on the basolateral surface of hepatocytes [15], [16], [23], [24], [25]. The only difference between the *P. gallinaceum* RII and the RII consensus, present in all *Plasmodium* species, is a single amino acid substitution, despite the fact that *P. gallinaceum* does not infect hepatocytes.

The repeat region is species-specific, immunodominant and constitutes about one half of the molecule. It contains multiple copies of tandemly arrayed repeat peptides (Figure 1) and has no obvious sequence homology to any known protein. The repeats contain B-cell immunodominant epitopes and antibodies specific to this region can protect against malaria by blocking sporozoite invasion of host hepatocytes [26], [27]. The function of the CSP repeat region within the parasite life cycle has so far not been investigated. However, there are reasons to believe that this large domain of CSP could perform perhaps multiple functions for the parasite. The repeat peptide structure might lend itself well to high avidity interactions with host receptors, for example GAGs which are composed of repeating sugar units. Multivalency is able to enhance the affinity of protein-carbohydrate interactions, particularly those involving GAGs [28], and the repeats could provide multivalent binding sites. In addition, the species-specific nature of the region may indicate a role in maintaining the host specificity of infection.

*P. berghei* parasites, in which the entire CS gene (*PbCS*) was replaced with its *P. gallinaceum* orthologue, failed to both invade salivary glands and to infect mice [29], although sporozoite development and CSP expression appeared unaffected. *P. gallinaceum* is carried by the mosquito vector *Aedes aegypti* whereas *P. berghei* is carried by anopheline vectors. The lack of infectivity with the *PgCS* replacement parasites could be due to the inability of PgCSP to bind to *A. stephensi* salivary gland receptors and rodent liver receptors.

To assess whether the repeat region has a role in salivary gland or vertebrate infectivity, we generated transgenic *P. berghei* parasites carrying the *PgCS* gene, in which the repeat region was replaced with the *PbCS* repeat region (Figure 1), in order to determine if introduction of the wild-type sequence rescues infectivity. Another parasite line containing the full-length *PbCS* N-terminal sequence (up to RI), the *PbCS* repeat region and the *PgCS* C-terminal sequence was also generated and allowed further analysis of the role of the N- and C-terminal regions (Figure 1).

## Results

### Generation of transgenic parasites

Transgenic *P. berghei* parasites were generated that carried chimeric *P. gallinaceum* - *P. berghei* CS genes. In the first transgenic parasite line, the endogenous *PbCS* gene was substituted with the *PgCS* gene in which the repeat region was replaced with the homologous region of *P. berghei*: line PgCS/Pb^RR^ (Figure 1A). A second line contained both the N-terminal region and repeat region of *P. berghei,* while the C-terminal region was replaced with the homologous sequence of *P. gallinaceum*: line PbCS/Pg^CT^ (Figure 1A). As controls, we generated: i) a transgenic line carrying the wild type *PbCS* gene and the drug selectable marker inserted in the *PbCS* locus: line PbCS^DHFR^ and ii) a transgenic line carrying the *PgCS* gene in which the *Spe*I and *Xho*I restriction sites had been introduced on either side of the repeat region: line PgCS^SX^ (Figure 1A). A schematic representation and alignment of PbCSP and the CS proteins expressed in the transgenic parasites, and their corresponding names, are presented in Figure 1A and 1B respectively.

**Figure 2:**
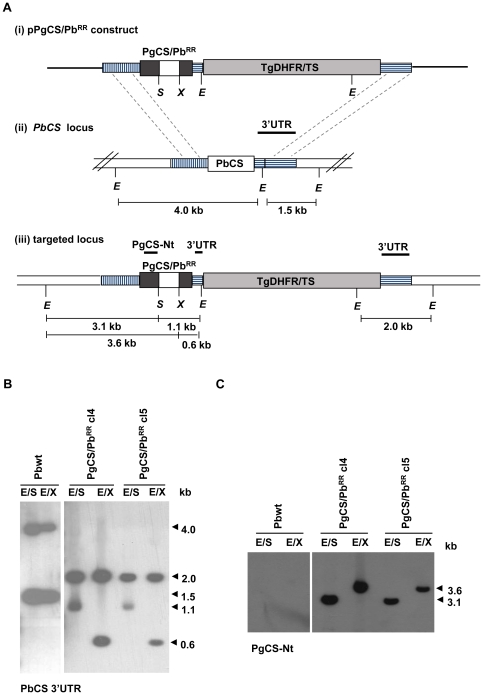
Generation and southern blot analysis of transgenic PgCS/Pb^RR^ parasite lines. (**A**) Schematic representation of (i) the PgCS/Pb^RR^ targeting construct, (ii) the wt *PbCS* locus and (iii) the targeted locus after recombination between the *PbCS* 5′UTR and 3′UTR sequences. The vertically dashed box indicates the 1.13 kb 5′UTR sequence used in the construct and the horizontally dashed boxes indicate the 0.3 kb and 0.85 kb 3′UTR sequences between which the TgDHFR-TS selectable marker cassette (light grey) was inserted in the construct. The PgCS/Pb^RR^ chimeric gene contains the *PgCS* N- and C-terminal regions (dark grey boxes) and the *PbCS* repeat region (white box) flanked by the *Spe*I (*S*) and *Xho*I (*X*) sites. In the wt PbCS locus the white box indicates the full *PbCS* gene. Thick black lines indicate the probes used in southern blots. E: *EcoR*V site. (**B**) Southern blot of *EcoR*V/*Spe*I (E/S) and *EcoR*V/*Xho*I (E/X) digested genomic DNA from Pbwt parasites and transgenic PgCS/Pb^RR^ parasites (clones 4 and 5), hybridised with the *PbCS* 3′UTR probe. Two bands of 4.0 and 1.5 kb were present in Pbwt DNA in both digestions. E/S digested DNA from the PgCS/Pb^RR^ clones revealed two bands of 1.1 and 2.0 kb, while E/X digested DNA revealed two bands of 0.6 and 2.0 kb, demonstrating the replacement of the endogenous *PbCS* gene with the targeted construct. (**C**) The same membrane was then hybridised with the *PgCS* N-terminal probe (PgCS-Nt), encompassing the entire N-terminal region of *PgCS*. No band was present in Pbwt DNA. However, a band of 3.1 kb or 3.6 kb was present in the E/S or E/X digested transgenic DNA, respectively.

The targeting constructs were designed to direct the double cross-over event between the 1.13 kb sequence of the *PbCS* 5′ untranslated region (UTR) and the 0.85 kb sequence of the *PbCS* 3′ UTR in the linearized plasmid and their corresponding sequences in the *PbCS* locus (Figure 2A and Figure S1A). Two targeting constructs, pPgCS/Pb^RR^ and pPgCS^SX^ were then used to transfect *P. berghei* wild type (Pbwt) parasites (Figure 2A and Figure S1A respectively).

In construct pPgCS^SX^ (Figure S1A), the *Spe*I and *Xho*I restriction sites had been introduced by site-directed mutagenesis into the *PgCS* sequence in order to mediate exchange of the *PgCS* repeat region with the homologous sequence of *P. berghei*. The *Spe*I site was inserted immediately downstream of the *PgCS* RI core sequence, NLNQP (Figure 1). The *Xho*I restriction site was inserted after the *PgCS* repeat region and before RII (Figure 1). Correct insertion of the construct, to generate the control transgenic parasite line PgCS^SX^ (clones 1 and 2), was confirmed by Southern blot analysis (Figure S1).

A second construct was generated by replacing the *PgCS* repeat region in pPgCS^SX^ with the *PbCS* repeat region, which had been amplified from genomic DNA. The resulting construct, pPgCS/Pb^RR^, was then tranfected into Pbwt parasites to create the transgenic line PgCS/Pb^RR^ (clones 4 and 5), and correct integration was confirmed by southern blot (Figure 2).

**Figure 3:**
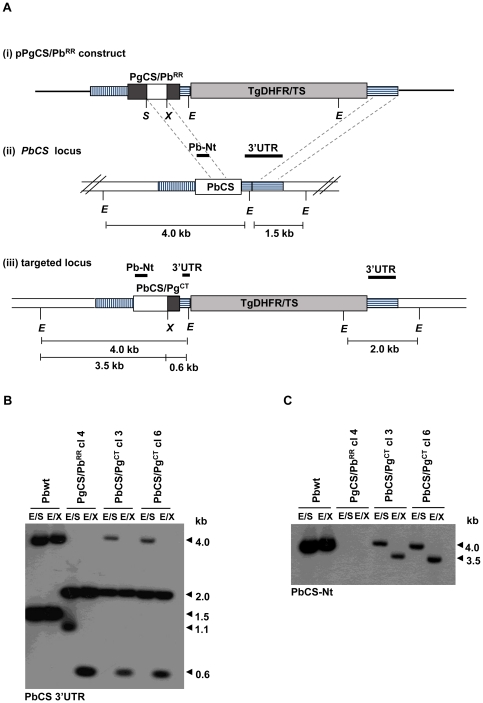
Generation and southern blot analysis of transgenic PbCS/Pg^CT^ parasite lines. (**A**) Schematic representation of (i) the PgCS/Pb^RR^ targeting construct, (ii) the wt *PbCS* locus and (iii) the targeted locus after recombination between the *PbCS* repeat region (white box) and the *PbCS* 3′UTR (horizontally dashed box). The PbCS/Pg^CT^ chimeric gene contains both the *PbCS* N- terminal and repeat regions (white boxes) and the *PgCS* C-terminal region (dark grey box). The light grey box indicates the TgDHFR-TS selectable marker cassette. Thick black lines indicate the probes used in southern blots. E = *EcoR*V site, S = *Spe*I site and X = *Xho*I site. (**B**) Southern blot of *EcoR*V/*Spe*I (E/S) and *EcoR*V/*Xho*I (E/X) digested genomic DNA from Pbwt parasites, transgenic PgCS/Pb^RR^ clone 4 parasites and PbCS/Pg^CT^ parasites (clones 3 and 6), hybridised with the *PbCS* 3′UTR probe. PgCS/Pb^RR^ parasites acted as a control for the presence of both the *Spe*I and *Xho*I sites in the CS chimeric gene. Both PbCS/Pg^CT^ clones contained a gene with the *Xho*I site but lacked the *Spe*I site, as demonstrated by the presence of the 4.0 and 2.0 kb bands after E/S digestion and the 2.0 and 0.6 kb bands after E/X digestion, indicating the 5′ cross-over event had occurred between the repeat regions. (**C**) Southern blot of E/S and E/X digested genomic DNA, hybridised with the *PbCS* N-terminal region probe. A band of 4.0 kb was detected in both the Pbwt DNA and E/S digested DNA from the PbCS/Pg^CT^ clones, demonstrating the presence of the PbCS N-terminal sequence in the transgenic parasites. Genomic DNA from the PbCS/Pg^CT^ clones, digested with E/X, revealed a band of 3.5 kb, indicating the presence of the *Xho*I site. No bands were visible in PgCS/Pb^RR^ transgenic parasite DNA due to the absence of the PbCS N-terminal sequence.

Other integration events were also observed after transfection with the pPgCS/Pb^RR^ vector, as a consequence of cross-over events that took place between sequences present in the internal part of the construct and the homologous sequences in the *PbCS* locus. This resulted in the generation of two further transgenic parasite lines, PbCS/Pg^CT^ and PbCS^DHFR^ (Figure 3 and Figure S2). PbCS/Pg^CT^ clones 3 and 6 originated from cross-over events at the 0.5 kb *PbCS* repeat region and the 3′ UTR sequence (Figure 3A). The PbCS/Pg^CT^ parasite line generated therefore contained a chimeric CS gene where both the N-terminal sequence and the repeat region were from *P. berghei,* while the C-terminal sequence was from *P. gallinaceum*, as demonstrated by Southern blot analysis (Figure 3).

Moreover, just 300 bp of the PbCS 3′ UTR, present between the chimeric CS gene and the TgDHFR/TS cassette in the construct pPgCS/Pb^RR^, was sufficient to direct recombination, generating the PbCS^DHFR^ transgenic line (Figure S2A). Double cross-over recombination, between the two *PbCS* 3′ UTR regions present in the construct and the homologous regions in the *PbCS* locus, resulted in the insertion of only the TgDHFR/TS cassette, in the 3′ UTR region of the *PbCS* locus, leaving the endogenous *PbCS* gene intact. The transgenic PbCS^DHFR^ clones 9 and 10 generated (Figure S2) were used as a control for any phenotypic effects arising from the presence of the selectable marker cassette within the CS locus.

The insertion events described, and the presence of the correct CS gene sequences in the transgenic parasites lines, was further confirmed by sequencing the CS genes, which had been PCR amplified from genomic DNA.

### Development of the transgenic parasites in the mosquito vector

Blood stage parasites from all transgenic lines replicated normally in mice and *in vitro* generated gametocytes that developed into fertile gametes. The development of the transgenic parasites in the mosquito was monitored in *Anopheles stephensi* fed on infected mice. Mosquito midguts infected with each of the transgenic clones, and dissected at 14 and 18 days post-infection (p.i.), revealed normal oocyst development compared to mosquitoes infected with Pbwt parasites. All transgenic oocysts had the same morphology as Pbwt oocysts. Interestingly, melanisation (observed in mosquitoes infected with PgCS-replacement *P. berghei* parasites [29]) was only seen in midguts infected with transgenic lines containing *PgCS* sequences. However, it occurred with a very low frequency both in terms of the proportion of infected midguts and also the number of melanised oocysts per midgut.

Midguts dissected on day 14 p.i. showed no significant differences in oocyst and sporozoite numbers between any of the transgenic parasite lines and Pbwt parasites (Table 1), indicating that both heterologous PgCSP and the chimeric proteins function normally during sporozoite ontogeny in the mosquito vector *A. stephensi*. However, at day 21 p.i., PgCS^SX^ parasites showed a significant reduction in the number of salivary gland sporozoites compared to both Pbwt and PbCS^DHFR^ control parasites, similar to the results previously reported for mosquitoes infected with PgCS-replacement *P. berghei* parasites [29]. Likewise, PgCS/Pb^RR^ parasites showed a striking and significant reduction in salivary gland sporozoites compared to Pbwt and PbCS^DHFR^ parasites (Table 1). Furthermore, no significant difference was observed between PgCS^SX^ and PgCS/Pb^RR^ clone 4 (p>0.09), although the difference between PgCS^SX^ and PgCS/Pb^RR^ clone 5 was significant (p<0.03). However, this result for PgCS/Pb^RR^ clone 5 may have been due to contaminating hemolymph sporozoites or a greater number of sporozoites adhering to the outside of the glands.

**Table 1:** Transgenic parasite development in the *Anopheles stephensi* mosquito vector.

Parasite	Midgut oocysts	Midgutsporozoites	Sporozoites/oocyst	Salivary gland sporozoites	Salivary gland sporozoites/oocyst
Pbwt	182±42	12,174±19,070	63±79	28,412±8,167	134±14
PbCS^DHFR^ cl9	195±105	26,021±40,551	144±217	25,626±5,185	154±70
PbCS^DHFR^ cl10	190±47	15,377±6,451	89±53	14,447±4,503	83±40
PgCS^SX^ cl1	189±93	6,447±5,221	36±27	297±336^a^	2±2
PgCS/Pb^RR^ cl4	202±66	14,844±6,773	86±69	922±697^a^	6±5
PgCS/Pb^RR^ cl5	194±32	13,892±8,871	68±37	1,276±300^a^^,^ b	7±3
PbCS/Pg^CT^ cl3	207±77	33,821±32,294	240±311	15,779±3,832	80±17
PbCS/Pg^CT^ cl6	248±55	32,520±22,484	133±99	18,291±8,231	78±47

a Significant difference compared to Pbwt and PbCS^DHFR^ transgenic parasites; p<0.01.

b Significant difference compared to PgCS^SX^; p<0.03.

*A. stephensi* mosquitoes were fed on mice infected with one of the transgenic clones or Pbwt parasites. Values represent the mean ± S.D. of at least three independent experiments, each with a minimum of 50 mosquitoes.

The low numbers of salivary gland sporozoites in mosquitoes infected with PgCS/Pb^RR^ parasites suggests that the replacement of the *PgCS* repeat region with the homologous *PbCS* repeat region is not sufficient to rescue salivary gland invasion. In contrast, the PbCS/Pg^CT^ transgenic parasites invaded the mosquito salivary glands normally. These parasites contain only the C-terminal sequence of PgCSP, which appears to function normally in *A. stephensi* salivary gland infection. The results suggest that the N-terminal region plays an important role in salivary gland infection, although it is still possible that repeat region residues co-operate with N-terminal residues in infectivity. This is in agreement with previous studies, which used recombinant N-terminal peptides to demonstrate a role for the region in salivary gland binding [13], [22].

### Infectivity of the transgenic sporozoites for the vertebrate host

To determine the infectivity of the transgenic parasites for the vertebrate host, salivary gland and midgut sporozoites, collected 21 days p.i., were injected intravenously into C57BL/6 mice. All mice injected with Pbwt salivary gland sporozoites developed a parasitaemia, with a pre-patent period ranging from 3.5 to 4.6 days for mice injected with 5,000 and 1,000 sporozoites respectively (Table 2). Interestingly, for the PbCS^DHFR^ transgenic parasites, only injection with 5,000 sporozoites resulted in a 100% infection rate, and the pre-patent period was longer, ranging from 5.3 to 6.5 days after injection of 5,000 or 1,000 PbCS^DHFR^ sporozoites, respectively. A 1 day delay in the pre-patent period indicates a 90% decrease in the infective inoculum [30]. This difference in infectivity between Pbwt and PbCS^DHFR^ parasites suggests a moderate effect on vertebrate host infection due to the presence of the selectable marker cassette in the CS locus.

**Table 2:** Infectivity of salivary gland sporozoites for the vertebrate host.

Parasite	Sporozoites injected	Miceinfected	Pre-patentperiod^a^
Pbwt	1,000	5/5	4.6
	3,000	3/3	4.0
	5,000	3/3	3.5
PbCS^DHFR^ cl9	1,000	1/6	6.0
	3,000	4/6	6.0
	5,000	4/4	5.5
PbCS ^DHFR^ cl10	1,000	2/4	6.5
	3,000	2/4	6.0
	5,000	3/3	5.3
PgCS^SX^ cl1	5,000	0/3	-
PgCS/Pb^RR^ cl4	5,000	0/5	-
PgCS/Pb^RR^ cl5	5,000	0/5	-
PbCS/Pg^CT^ cl3	1,000	0/3	-
	3,000	3/4	5.7
	5,000	5/6	5.5
PbCS/Pg^CT^ cl6	1,000	1/3	6.0
	3,000	3/4	6.0
	5,000	4/5	5.5

a Pre-patent period is the number of days between injection and first appearance of the parasites in the peripheral blood.

C57BL/6 mice were injected intravenously with different numbers of Pbwt or transgenic sporozoites collected on day 21 p.i. from *A. stephensi* salivary gland preparations. The number of mice that became infected and the pre-patent period were both recorded.

Mice injected with 5,000 sporozoites, from salivary gland preparations of either PgCS^SX^ or PgCS/Pb^RR^ infected mosquitoes, did not develop a parasitaemia, demonstrating that the replacement of the *PgCS* repeat region with the homologous wildtype *PbCS* repeat region is also unable to rescue vertebrate infectivity.

The PbCS/Pg^CT^ clones had very similar infection rates and pre-patent periods compared to the PbCS^DHFR^ clones, indicating that the N-terminal sequence is also important for vertebrate infectivity. Moreover, the PgCSP C-terminal sequence appears to function efficiently in vertebrate infectivity, despite the fact that only 39 out of the 85 amino acids in the C-terminus of CSP are conserved between *P. berghei* and *P. gallinaceum,* including 10 of the 18 Region II residues (Figure 1B).

We also investigated the infectivity of midgut sporozoites in C57BL/6 mice. Only one out of two mice injected with 500,000 Pbwt sporozoites became parasitaemic, with a pre-patent period of 6.0 days (Table 3). This is consistent with the known lesser infectivity of midgut sporozoites compared to salivary gland sporozoites [31]. A pre-patent period between 7.0 and 8.0 days was observed after injection of 500,000 PbCS^DHFR^ or PbCS/Pg^CT^ sporozoites, demonstrating a similar delay compared to Pbwt parasites as that observed after the injection of salivary gland preparation sporozoites. PgCS/Pb^RR^ and PgCS^SX^ midgut sporozoites never infected mice (Table 3).

**Table 3:** Infectivity of midgut sporozoites for the vertebrate host.

Parasite	Sporozoites injected	Mice infected	Pre-patent period^a^
Pbwt	100,000	0/3	-
	300,000	2/4	7.0
	500,000	1/2	6.0
PbCS^DHFR^ cl9	500,000	1/4	8.0
PbCS^DHFR^ cl10	500,000	1/2	7.0
PgCS^SX^ cl1	500,000	0/5	-
PgCS/Pb^RR^ cl4	500,000	0/4	-
PgCS/Pb^RR^ cl5	500,000	0/4	-
PbCS/Pg^CT^ cl3	500,000	1/5	8.0
PbCS/Pg^CT^ cl6	500,000	2/5	7.0

a Pre-patent period is the number of days between injection and first appearance of the parasites in the peripheral blood.

C57BL/6 mice were injected intravenously with Pbwt or transgenic midgut sporozoites collected on day 21 p.i.. The number of mice that became infected and the pre-patent period were both recorded.

### CSP expression in transgenic parasite lines

**Figure 4:**
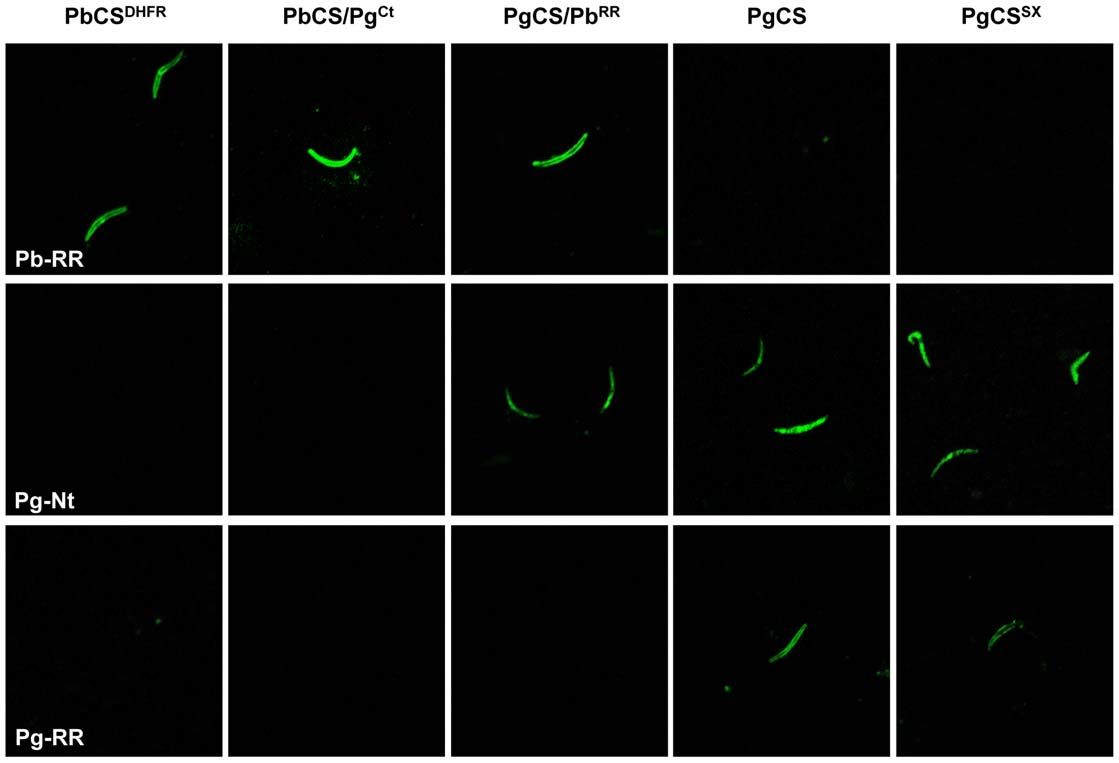
Sporozoite CSP expression. Confocal immunofluorescence microphotographs of transgenic midgut sporozoites incubated at room temperature and developed with either a monoclonal antibody directed against the PbCSP repeat region (Pb-RR), a serum directed against the PgCSP N-terminal region (Pg-Nt), or a serum directed against the PgCSP repeat region (Pg-RR). Antibodies against the PbCSP and PgCSP repeat regions showed surface expression of the CSPs, whereas antibody against the PgCSP N-terminal region revealed mainly an intracellular pattern of expression.

Immunofluorescence assays (IFA) were carried out to analyse both CSP expression and gliding motility of midgut sporozoites, since PgCS/Pb^RR^ and PgCS^SX^ transgenic parasites do not appear to invade the salivary glands. Freshly dissected sporozoites were spotted onto a glass slide and either left at room temperature (RT) or placed at 37°C for 30 minutes to induce gliding motility [32]. The parasites were then allowed to react with either a monoclonal antibody directed against the PbCSP repeat region, a polyclonal antibody directed against the PgCSP N-terminal region, or a polyclonal antibody directed against the PgCSP repeat region (Figures 4 and S3).

Sporozoites from each of the transgenic clones, except PgCS^SX^ and PgCS replacement parasites, showed a clear and uniform surface staining when incubated with the antibody against the PbCSP repeat region (Figure 4), similar to the staining observed for Pbwt sporozoites [33]. Although midgut sporozoites display limited gliding motility compared to salivary gland sporozoites, after incubation at 37°C with the PbCSP repeat region antibody, trails could be observed with Pbwt, PbCS^DHFR^, PbCS/Pg^CT^ and PgCS/Pb^RR^ parasites at a similar frequency, between 5 and 10% (Figure S3). However, the trails were extremely variable in shape and length and only very rarely were circular trails (<1% of total trails), similar to those seen for salivary gland sporozoites, observed (Figure S4). These results demonstrated that the presence of lengthy heterologous *PgCS* sequences in the chimeric CS genes does not significantly modify the surface expression of the proteins or the parasites' gliding motility. Therefore, the inability of the PgCS/Pb^RR^ parasites to infect the mosquito salivary glands or the vertebrate host does not appear to be due to incorrect CSP surface expression or motility.

Interestingly, analysis of the PgCS/Pb^RR^, PgCS^SX^ and PgCS replacement parasites with the PgCSP N-terminal region antiserum revealed the localization of the CSP to be mainly intracellular, throughout the cytosol (Figure 4). In contrast, PgCS/Pb^RR^ parasites, when analysed with the antibody against the PbCSP repeat region, displayed clear surface CSP expression (Figure 4) [33]. Similarly, PgCS^SX^ and PgCS replacement sporozoites, incubated with a serum against the PgCSP repeat region, revealed normal surface staining (Figure 4), and also the presence of clumps of immunoreactive material either outside the parasite body or in proximity to its surface, following incubation at 37°C (Figure S3) [29], indicating that the introduction of the two restriction endonuclease sites in the *PgCS* sequence had no obvious effect on protein expression and release.

These IFA results are in agreement with recent data that report the absence of the N-terminal CSP sequence on the surface of *P. berghei* oocyst sporozoites [34]. The two antibodies are therefore reacting with different processed forms of CSP: the repeat region antibodies with a lower molecular weight (MW) form, lacking the N-terminal region and associated with the surface of the sporozoite, and the PgCSP N-terminal region serum with a higher MW form present in the cytoplasm.

Salivary gland sporozoites from Pbwt, PbCS^DHFR^ and PbCS/Pg^CT^ infected mosquitoes, incubated with the PbCSP repeat region antibody, displayed a regular CSP surface expression and the characteristic circular gliding motility (Figure S4), with no differences observed either in the frequency of motile sporozoites or the percentage forming trails of at least one complete loop.

### CSP processing in transgenic parasite lines

**Figure 5:**
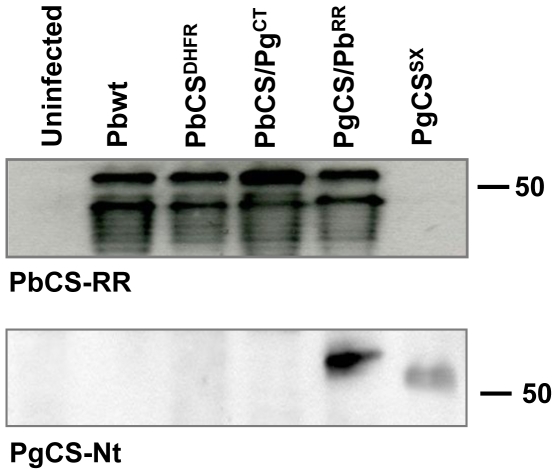
Western blot analysis of CSP expression and processing in transgenic midgut sporozoites 18 days post-infection. CSP expression was revealed by incubation with either a monoclonal antibody directed against the PbCSP repeat region (PbCS-RR) or a serum directed against the PgCSP N-terminal region (PgCS-Nt). As a control, midgut lysates from uninfected mosquitoes were also analysed. Antibody against the PbCSP repeat region reveals a higher molecular weight precursor polypeptide and a lower molecular weight processed polypeptide. A ladder of degradation products is also visible. Antibody against the PgCSP N-terminal region reveals only the higher molecular weight protein.

We investigated the expression and processing of CSP in the transgenic parasites by Western blot analysis of midgut sporozoite lysates collected 18 days p.i.. The PbCSP repeat region antibody revealed both the high and the low MW CSP forms in PbCS^DHFR^, PbCS/Pg^CT^ and PgCS/Pb^RR^ transgenic parasites (Figure 5) [11], [35]. A ladder of bands, weaker in intensity and ranging in size between ∼30 and 46 kDa, was also present and has previously been described [36], [37]. These results demonstrate that the CSPs in the transgenic parasite lines are expressed and processed similarly to CSP in Pbwt parasites.

Incubation with the PgCSP N-terminal region antiserum revealed only the high MW form, corresponding to the CS precursor, in the PgCS/Pb^RR^ and PgCS^SX^ parasite lines, in agreement with N-terminal region cleavage to yield the mature, processed form of CSP, containing the repeat region and C-terminal region. The band present in the PgCS/Pb^RR^ parasites appears to be slightly bigger than the expected ∼54 kDa band (revealed after incubation with the PbCSP repeat region antibody). This difference in size is likely due to the fact that the PbCSP repeat region is extremely proline rich and this has been shown to be responsible for anomalous migration patterns of proteins in SDS-PAGE [38].

## Discussion

The aim of this work was to investigate the role of the CSP repeat region and the amino terminal region in the recognition of species-specific vector and host receptors. In previous studies, transgenic *P. berghei* parasite lines, in which either the entire endogenous CS gene or the repeat region was replaced with the *P. falciparum* orthologous sequence, both showed a ten fold reduction in *A. stephensi* salivary gland invasion [27], [29], suggesting a functional role of the repeat sequence in the recognition of species-specific host receptors. Our results indicate that the repeat region, on its own, is unlikely to represent the sporozoite species-specific salivary gland or hepatocyte ligand, because the replacement of the *PgCS* repeat region with the *PbCS* repeat region in the PgCS/Pb^RR^ transgenic parasites doesn't rescue sporozoite infectivity of either *A. stephensi* salivary glands or vertebrate hepatocytes. However, it is still possible that sequences in the N-terminal region and repeat region cooperate in, and are both required for, sporozoite infectivity.

The *P. berghei* transgenic parasites bearing a chimeric CSP displaying the *P. falciparum* CS repeat region, also had the RI motif and 14 residues upstream (RI-plus) exchanged with those of *P. falciparum* [27]. The change in either the repeat region or RI-plus could have been responsible for the 10-fold decrease in salivary gland invasion observed with these parasites. Although the RI core is the same between the two species (KLKQP), RI-plus differs between *P. berghei* and *P. falciparum*. In both *P. falciparum* and *P. berghei* nine of the fourteen residues are charged, indicating that the regions might be involved in interactions with host receptors. Nevertheless, it is possible that the repeat region has a function in infectivity acting in concert with other CSP regions. The CSP repeat region may fulfill a structural role, with the repeats from neighboring CSP molecules interlocking to form a rigid sheath surrounding the parasite [39]. Proline-rich tandem repeats generally form extended structures and flexible regions in proteins, and have been shown to function both as structural elements and in binding processes [40].

PbCS/Pg^CT^ parasites, containing both the *PbCS* N-terminal and repeat regions, were not impaired in salivary gland or vertebrate infectivity, pointing to a role for the N-terminal region in infection. Indeed, sera against the *P. falciparum* and *P. berghei* CSP N-terminal regions have been shown to inhibit sporozoite invasion of hepatocytes *in vitro* and HepG2 cells [41]. In previous investigations, a role in salivary gland infectivity was implied based on binding inhibition experiments, in which specific peptides were analysed for their ability to block recombinant CSP and sporozoite binding to the glands [13], [22]. However, the results of peptide inhibition studies can be due to steric hindrance. Therefore, gene targeting provides a more reliable system for determining the function of protein domains.

IFA analysis with a serum directed against the PgCSP N-terminal region appeared to localise the PgCS/Pb^RR^ and PgCS^SX^ proteins mainly to the intracellular compartment, in contrast to antibodies against the PbCSP and PgCSP repeat regions, which revealed fluorescence at the surface of transgenic sporozoites (Figure 4). The results indicate that two forms of CSP are present in our transgenic lines: a full length protein localised intracellularly and a processed form, lacking the N-terminal region, and expressed uniformly on the sporozoite surface. This suggests possibly an intracellular site of CSP processing or alternatively, the protein may be cleaved either during its release from the apical organelles, before translocation onto the sporozoite surface, or after contact with target cells. Contradictory results support both intracellular [35], [42], [43] and surface [44], [45] sites of CSP cleavage. In *Toxoplasma gondii*, the N-terminal region of the adhesive protein, MIC2, is trimmed prior to secretion [46]. Another *T. gondii* protein, MIC3, which is re-localised to the surface of the parasite during invasion, is synthesised with an N-terminal propeptide that is cleaved during trafficking to the microneme. The processing was shown to be required for the protein to function as an adhesin [47] and it is possible that a similar mechanism operates in *Plasmodium* CSP.

Western blot analysis demonstrated that at least a large part of the N-terminal region is cleaved from the precursor polypeptide in each of the transgenic lines. Furthermore, data suggests that the protease that mediates CSP cleavage is parasite derived rather than host derived [45], which is compatible with an intracellular site of processing. The protease may therefore differ between *P. berghei* and *P. gallinaceum* parasites, which could in turn affect the correct processing of the transgenic CS proteins. However, the results indicate that the *P. berghei* protease is able to similarly cleave PbCSP and the chimeric CS proteins.

Our results demonstrate that the N-terminal region of CSP is important for infectivity, which suggests that at least part of this sequence is likely to be present on the sporozoite surface, possibly acting as a ligand for host receptors. This observation is partially in agreement with the recent results from Coppi et al. [34], in which they identify a higher MW CSP on the surface of the sporozoites. We have tried to determine the precise cleavage site in our transgenic lines by sequencing the low MW form of CSP, however we were unable to obtain enough material.

Recent results indicate that RI plays a critical role in CSP processing either because it is required for efficient cleavage or because it contains the cleavage site [34]. RI in *P. gallinaceum* (NLNQP) differs from mammalian *Plasmodium spp.* RI (KLKQP) and yet the PgCS/Pb^RR^ protein is still processed, probably by the same proteolytic enzyme. Both the high and low MW CSP bands of the PgCS/Pb^RR^ parasites run slightly higher compared to the equivalent bands of the PbCS/Pg^CT^ parasites in Western blot analysis (Figure 5). This is in part due to the fact that the chimeric proteins have different sizes: PgCS/Pb^RR^ and PbCS/Pg^CT^ contain 382 and 347 amino acids respectively. If the cleavage site is located at RI, one would expect the low MW bands from these two parasite lines to be the same size, since the processed CSPs in both these lines would contain the PbCS repeat region and the PgCS C-terminal region. Only the high MW bands, representing the precursor polypeptides, would differ in size. However, in our Western blot analysis, both the high and low MW bands from the PgCS/Pb^RR^ parasites appear to have larger MWs compared to the equivalent bands in the PbCS/Pg^CT^ parasites. This could be explained if the cleavage site is located upstream of RI, in the N-terminal region: an extra 25 amino acids are present just upstream of RI in the PgCSP sequence compared to the PbCSP sequence (Figure 1A), and this could explain the greater MW of the processed protein in PgCS/Pb^RR^ parasites compared to that of PbCS/Pg^CT^ parasites.

The sequence just upstream of RI contains a stretch of charged residues that, if included in the processed form of the protein, may act as a ligand. A peptide from PfCSP, spanning a region just upstream of RI (AA 93 to 113), was found to serve as a ligand for both hepatocytes [21] and salivary glands [13], [22]. Importantly, recognition of the short N-terminal peptide (PfCSP_65–110_) by sera from children living in a malaria-endemic region was associated with protection from disease [41]. The sequence of both RI and RI-plus varies considerably between *P. berghei* and *P. gallinaceum* (Figure 1A). In PbCSP, RI and the first 25 residues upstream of it contain 14 charged residues compared to the corresponding PgCSP sequence that contains only seven charged residues and this may explain the difference in infectivity observed between the PgCS/Pb^RR^ and the PbCS/Pg^CT^ parasites.

The numerous investigations into the functions of CSP's conserved motifs, RI and RII, have by no means led to a broad understanding of CSP's interactions during host infection. More recent work on CSP has focused on the N-terminal region of the protein [41] and the stretches of positively charged residues found throughout this region [19], [22]. The present investigation highlights, *in vivo*, a role for N-terminal residues in sporozoite infectivity in both the vertebrate and invertebrate hosts. These parasites could also provide a valuable rodent model to investigate the emerging role of the CSP N-terminal region in the induction of protective immune responses.

## Materials and Methods

### Ethics statement

Animal work conducted at Imperial College was performed according to UK Home Office Regulations and approved under Home Office License PPL 70/6453. Studies carried out at Perugia University have been performed according to the D.L 27 January 1992, n. 116, Italian legislation, and approved under Ethics Committee Licence n. PR 0161.

### DNA constructs

The targeting constructs pPgCS^SX^, pPgCS/Pb^RR^ and pPbCS^DHFR^ generated in this study contained the following structural elements: (i) the first 1,130 nucleotides of the *PbCS* 5′ UTR sequence immediately upstream of the start codon; (ii) PgCS, PbCS or a chimeric form of the CS coding sequence and (iii) the first 1,150 nucleotides of the *PbCS* 3′ UTR sequence immediately downstream of its stop codon, in which the *T. gondii* dihydrofolate reductase/thymidylate synthase (TgDHFR/TS) drug selectable marker cassette (5,150 bp) was inserted at its *Hind*III site.

All three constructs were generated from a targeting construct, pPgCS, already available in our laboratory which contains the full *PgCS* coding sequence [29]. The *Spe*I and *Xho*I sites were introduced on either side of the *PgCS* repeat region by PCR based site-directed mutagenesis. Primers used to introduce *Spe*I were: For1 5′-GAGAAAATGTTGTGAATCTTAATCAACCAACTAGTGTTGGAGGAAATGGTGGTGTTCAACCTGCTG-3′ (*Spe*I is underlined) and Rev1 complementary to the forward primer. Primers used to introduce *Xho*I were: For2 5′-CTGAAGAAGAAAAGGAGGATGAACCAATACCAGATCTCGAGCCAACTCAAGAAGAAATAGATAAATATTTAAAAAG-3′ (*Xho*I is underlined) and Rev2 complementary to the forward primer.

The 1.02 kb *PbCS* gene was amplified from *P. berghei* genomic DNA (Anka strain, clone 2.34) and sequenced. Forward and reverse primers were designed to amplify the *PbCS* repeat region from this sequence, introducing the *Spe*I and *Xho*I sites at either end of the repeat region (*Spe*for 5′-TAATAATAAATTGAAACAACCAACTAGTCCACCACCACCAAACCCAAATG-3′ (*Spe*I is underlined) and *Xho*Rev 5′-CCGCTCGAGGATATAAGAATCGTCATTATTATTATTTTTGTTATTG-3′ (*Xho*I is underlined)). The *PgCS* repeat region was then replaced with the *PbCS* repeat region, via *Spe*I and *Xho*I digestions. Sequencing analysis and alignment with the wild type *P. berghei* (GenBank: M14145) or *P. gallinaceum* (GenBank: 65959) sequences confirmed the correct CS genes inserted in each construct.

### Parasite transformation

Plasmids pPgCS^SX^ and pPgCS/Pb^RR^ were digested with *Apa*I to release the ∼8.6 kb targeting insert from the plasmid backbone and the insert was purified by gel electrophoresis. Purified schizonts of the *P. berghei* ANKA strain (clone 2.34) were transformed with 5 µg of targeting DNA using an Amaxa Gene Pulser set at program U33. Mutant parasites were obtained by the standard method of drug (pyrimethamine) selection in mice [48]. Pyrimethamine resistant parasites were subsequently cloned by limiting dilution.

### Southern blot analysis

Genomic DNA was isolated from parasites as previously described [33]. 1–3 µg of genomic DNA was digested with *Eco*RV alone or in combination with either *Spe*I or *Xho*I, separated on an agarose gel and blotted onto a nylon Hybond-N+ membrane (Amersham). The following DNA fragments were used as probes: (a) the 1.15 kb fragment of the *PbCS* 3′ UTR from the targeting constructs, (b) the entire N-terminal sequence of the *PbCS* coding region, (c) the entire N-terminal sequence of the *PgCS* coding region and (d) a 520 bp fragment amplified from the Tg*DHFR/TS* coding sequence with the primers TgFor123 (5′-AGAGGGGCATCGGCATCAAC-3′) and TgRev124 (5′-TTGAAAGAATGTCATCTCCG-3′). All hybridization experiments were carried out as previously described [33], [49].

### Sequencing of genomic DNA

The primers PbCS5′UTR3′F (5′-CCCTCACATAAGACAATCC-3′) and PbCS3′UTR5′R (5′-GTTTACACACGTCATATGTATG-3′), which anneal to the 5′ and 3′ UTR sequences of the CS locus, were used to amplify the full length CS gene from blood stage transgenic parasite genomic DNA, isolated from sporozoite-infected mice where possible. The PCR products were then sequenced directly (MWG-Biotech).

### Parasite development in the mosquito

Female mosquitoes (4–6 days old) of *A. stephensi* (strain sd500), were fed for 30 minutes on either wild type (wt) or transgenic parasite infected BALB/c mice (six- to eight-week-old female Balb/c from Harlan Sprague) with a parasitaemia ranging from 5% to 10%. Mosquitoes were dissected at days 14 and 21 post infection (p.i.) to determine the presence of oocysts and the number of parasites in the midguts and salivary glands.

### Parasite infectivity in mice

Midgut and salivary gland sporozoites were diluted with RPMI and intravenously (i.v.) injected into C57BL/6 mice (six- to eight-week-old female C57BL/6 from Charles River) in order to assess vertebrate infectivity. After sporozoite injection, blood samples were withdrawn from the tails at regular intervals up to 15 days p.i. and a minimum of 10,000 erythrocytes were examined on Giemsa stained blood smears. The pre-patent period was determined as the number of days between sporozoite injection and when parasites were first detected in the blood.

### Western blot analysis

Total proteins were extracted from infected (day 18 p.i.) and uninfected *A. stephensi* mosquito midguts using reducing sample buffer and separated on a 12% gel by SDS-PAGE. Blots were incubated for 1 hour at RT with the appropriate primary antibody: either a 1∶200 dilution of the monoclonal antibody 3D11 raised in mice against the PbCSP repeat region [11] or a 1∶200 dilution of a mouse serum raised against the PgCSP N-terminal region (a gift from Dr. Dharmendar Rathore), then developed with the enhanced chemiluminescence detection system, according to the manufacturer's instructions (Amersham Pharmacia).

### Immunofluorescence analysis

Freshly dissected sporozoites collected at day 21 p.i. from mosquito midguts and salivary glands were diluted in RPMI 1640 and spotted onto multiwell microscope slides. The slides were either incubated at 37°C, to induce sporozoite gliding [32], or at RT for 30 minutes. Parasites were fixed with 1% formaldehyde in 1× PBS and blocked with 1% BSA in 1× PBS for 30 minutes at RT. The slides were then incubated with one of three primary antibodies: the monoclonal antibody against the PbCSP repeat region [11], the serum against the PgCSP N-terminal region or a 1∶1000 dilution of a rabbit serum raised against the PgCSP repeat region. Antibody bound to the parasites or their trails was revealed by incubation with fluorescein isothiocyanate (FITC)-conjugated secondary antibody (Becton Dickinson). Analysis of expression was carried out using a Leica TCS SP 2 confocal microscope.

## Supporting Information

Figure S1 **Generation and southern blot analysis of transgenic PgCS^SX^ parasite lines.** (**A**) Schematic representation of (i) the PgCS**^SX^** targeting construct, (ii) the wt *PbCS* locus and (iii) the targeted locus after recombination between the *PbCS* 5′UTR and 3′UTR sequences. The vertically dashed box indicates the 1.13 kb 5′UTR sequence used in the construct and the horizontally dashed boxes indicate the 0.3 kb and 0.85 kb 3′UTR sequences between which the TgDHFR/TS selectable marker cassette (light grey) was inserted in the construct. The PgCS**^SX^** gene contains the full *PgCS* coding sequence (dark grey) into which the *Spe*I (*S*) and *Xho*I (*X*) sites were inserted on either side of the repeat region. Thick black lines indicate the probes used in southern blots. E = *EcoR*V. (**B**) Southern blot of *EcoR*V digested genomic DNA from Pbwt parasites and PgCS-replacement parasites (clone 14, [29]), that acted as negative and positive controls respectively, and transgenic PgCS**^SX^** parasites (clones 1 and 2). The blot was first hybridised with the PbCS 3′UTR probe, containing the full 1.15 kb fragment of the PbCS 3′UTR sequence present in the targeting construct. The 3′UTR probe hybridised with bands of 4.0 and 1.5 kb in digested Pbwt DNA and with bands of 4.2 and 2.0 kb in the transgenic parasite DNA. The size shift from 4.0 to 4.2 kb is due to the longer *PgCS* coding sequence. The 2.0 kb band indicates the presence of the TgDHFR/TS cassette in the PbCS locus. The membrane was also hybridized with probes encompassing the *TgDHFR/TS* gene (**C**), the *PbCS* gene (**D**) and the *PgCS* gene (**E**). The *TgDHFR/TS* probe revealed a band of 4.6 kb only in transgenic DNA (C). The *PbCS* probe revealed the 4.0 kb band only in the Pbwt DNA (D) while the *PgCS* probe bound to the correct 4.2 kb band only in the transgenic parasite DNA (E).
(TIF)

Figure S2 **Generation and southern blot analysis of transgenic PbCS^DHFR^ parasite lines.** (**A**) Schematic representation of (i) the PgCS/Pb^RR^ targeting construct, (ii) the wt *PbCS* locus and (iii) the targeted locus after recombination between the two PbCS 3′ UTR sequences (horizontally dashed boxes) flanking the TgDHFR-TS selectable marker cassette (light grey box). The targeted locus therefore carried the endogenous wt *PbCS* gene (white box) and, inserted in the 3′UTR, the TgDHFR-TS selectable marker cassette. Thick black lines indicate the probes used in southern blots. E: *EcoR*V site, S: *Spe*I site and X: *Xho*I site. (**B**) Southern blot of *EcoR*V/*Spe*I digested genomic DNA from Pbwt parasites and transgenic PbCS^DHFR^ parasites (clones 9 and 10), hybridised with the PbCS 3′ UTR probe. (**C**) Southern blot of *EcoR*V/*Xho*I digested genomic DNA hybridised with the PbCS N-terminal probe. Neither clone contained the *Spe*I or *Xho*I sites, as demonstrated by the presence of the 4.0 kb band in all lanes, indicating recombination had occurred between the short 0.3 kb PbCS 3′ UTR sequence upstream of the marker cassette. A second band in the transgenic parasite DNA of 2.0 kb, compared to the 1.5 kb band in the Pbwt DNA, indicated the insertion of the TgDHFR/TS cassette in the *PbCS* locus.
(TIF)

Figure S3 **Sporozoite CSP expression and motility.** Confocal immunofluorescence microphotographs of *P. berghei* wt and transgenic midgut sporozoites incubated at 37°C to induce motility and developed with either a monoclonal antibody directed against the PbCSP repeat region (Pb-RR), a serum directed against the PgCSP N-terminal region (Pg-Nt), or a serum directed against the PgCSP repeat region (Pg-RR). Sporozoites shed trails of material recognised by antibody against the PbCSP repeat region. Antibody against the PgCSP N-terminal region (Pg-Nt) revealed mainly an intracellular pattern of expression, and no trails, for the PgCS/Pb^RR^, PgCS^SX^ and PgCS replacement parasites. The antibody against the Pg-RR revealed the presence of clumps of immunoreactive material either outside the parasite body or in proximity to its surface.
(TIF)

Figure S4 **Salivary gland sporozoite CSP expression and motility.** Confocal immunofluorescence microphotographs of *P. berghei* wt and transgenic salivary gland sporozoites incubated at either room temperature (RT) or 37°C and developed with an antibody directed against the PbCSP repeat region (Pb-RR). The antibody revealed surface expression in PbCS^DHFR^ and PbCS/Pg^CT^ salivary gland sporozoites, similar to *P. berghei* wt salivary gland sporozoites. Sporozoites incubated at 37°C shed trails of material recognised by the anti-PbCSP repeat region antibody.
(TIF)
