# Supplementary figures and images for: Roles of the Amino Terminal Region and Repeat Region of the Plasmodium berghei Circumsporozoite Protein in Parasite Infectivity
Source: PLoS One. 2012 Feb 29;7(2):e32524. doi: 10.1371/journal.pone.0032524 (PMC3290588; doi:10.1371/journal.pone.0032524)

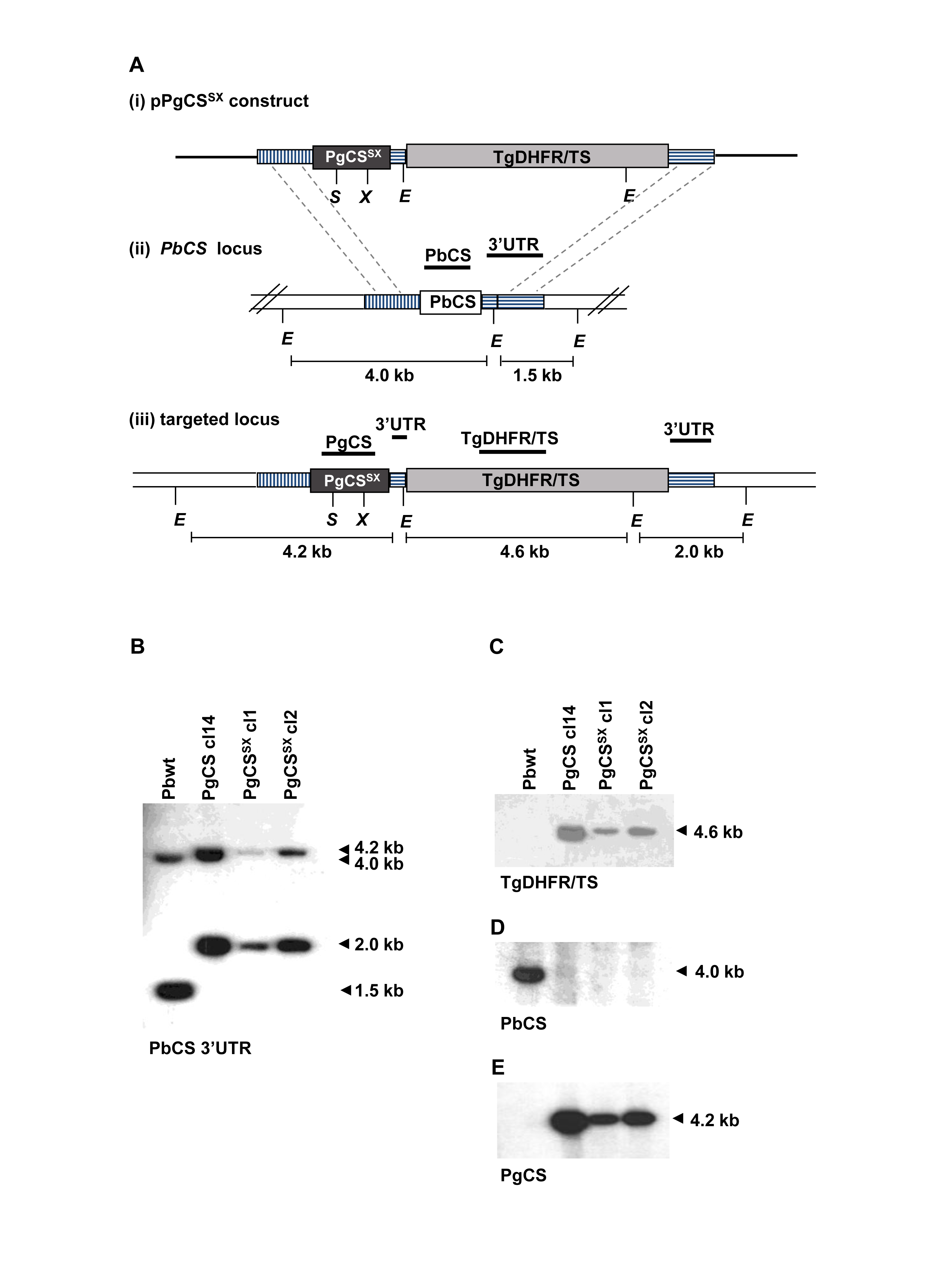

Supplement: Figure S1 — Generation and southern blot analysis of transgenic PgCSSX parasite lines. (A) Schematic representation of (i) the PgCSSX targeting construct, (ii) the wt PbCS locus and (iii) the targeted locus after recombination between the PbCS 5′UTR and 3′UTR sequences. The vertically dashed box indicates the 1.13 kb 5′UTR sequence used in the construct and the horizontally dashed boxes indicate the 0.3 kb and 0.85 kb 3′UTR sequences between which the TgDHFR/TS selectable marker cassette (light grey) was inserted in the construct. The PgCSSX gene contains the full PgCS coding sequence (dark grey) into which the SpeI (S) and XhoI (X) sites were inserted on either side of the repeat region. Thick black lines indicate the probes used in southern blots. E = EcoRV. (B) Southern blot of EcoRV digested genomic DNA from Pbwt parasites and PgCS-replacement parasites (clone 14, [29]), that acted as negative and positive controls respectively, and transgenic PgCSSX parasites (clones 1 and 2). The blot was first hybridised with the PbCS 3′UTR probe, containing the full 1.15 kb fragment of the PbCS 3′UTR sequence present in the targeting construct. The 3′UTR probe hybridised with bands of 4.0 and 1.5 kb in digested Pbwt DNA and with bands of 4.2 and 2.0 kb in the transgenic parasite DNA. The size shift from 4.0 to 4.2 kb is due to the longer PgCS coding sequence. The 2.0 kb band indicates the presence of the TgDHFR/TS cassette in the PbCS locus. The membrane was also hybridized with probes encompassing the TgDHFR/TS gene (C), the PbCS gene (D) and the PgCS gene (E). The TgDHFR/TS probe revealed a band of 4.6 kb only in transgenic DNA (C). The PbCS probe revealed the 4.0 kb band only in the Pbwt DNA (D) while the PgCS probe bound to the correct 4.2 kb band only in the transgenic parasite DNA (E). (TIF) [file pone.0032524.s001.tif]

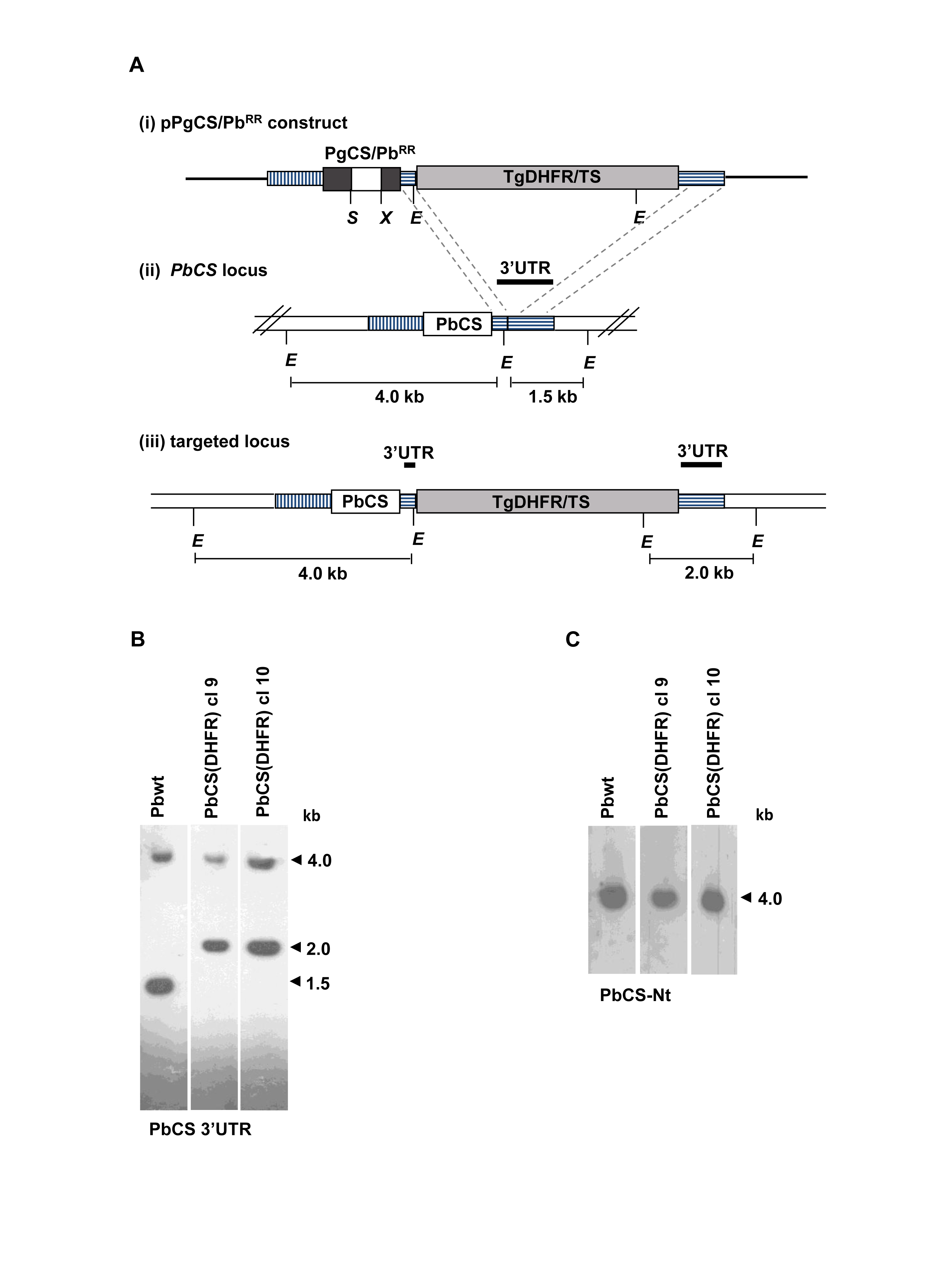

Supplement: Figure S2 — Generation and southern blot analysis of transgenic PbCSDHFR parasite lines. (A) Schematic representation of (i) the PgCS/PbRR targeting construct, (ii) the wt PbCS locus and (iii) the targeted locus after recombination between the two PbCS 3′ UTR sequences (horizontally dashed boxes) flanking the TgDHFR-TS selectable marker cassette (light grey box). The targeted locus therefore carried the endogenous wt PbCS gene (white box) and, inserted in the 3′UTR, the TgDHFR-TS selectable marker cassette. Thick black lines indicate the probes used in southern blots. E: EcoRV site, S: SpeI site and X: XhoI site. (B) Southern blot of EcoRV/SpeI digested genomic DNA from Pbwt parasites and transgenic PbCSDHFR parasites (clones 9 and 10), hybridised with the PbCS 3′ UTR probe. (C) Southern blot of EcoRV/XhoI digested genomic DNA hybridised with the PbCS N-terminal probe. Neither clone contained the SpeI or XhoI sites, as demonstrated by the presence of the 4.0 kb band in all lanes, indicating recombination had occurred between the short 0.3 kb PbCS 3′ UTR sequence upstream of the marker cassette. A second band in the transgenic parasite DNA of 2.0 kb, compared to the 1.5 kb band in the Pbwt DNA, indicated the insertion of the TgDHFR/TS cassette in the PbCS locus. (TIF) [file pone.0032524.s002.tif]

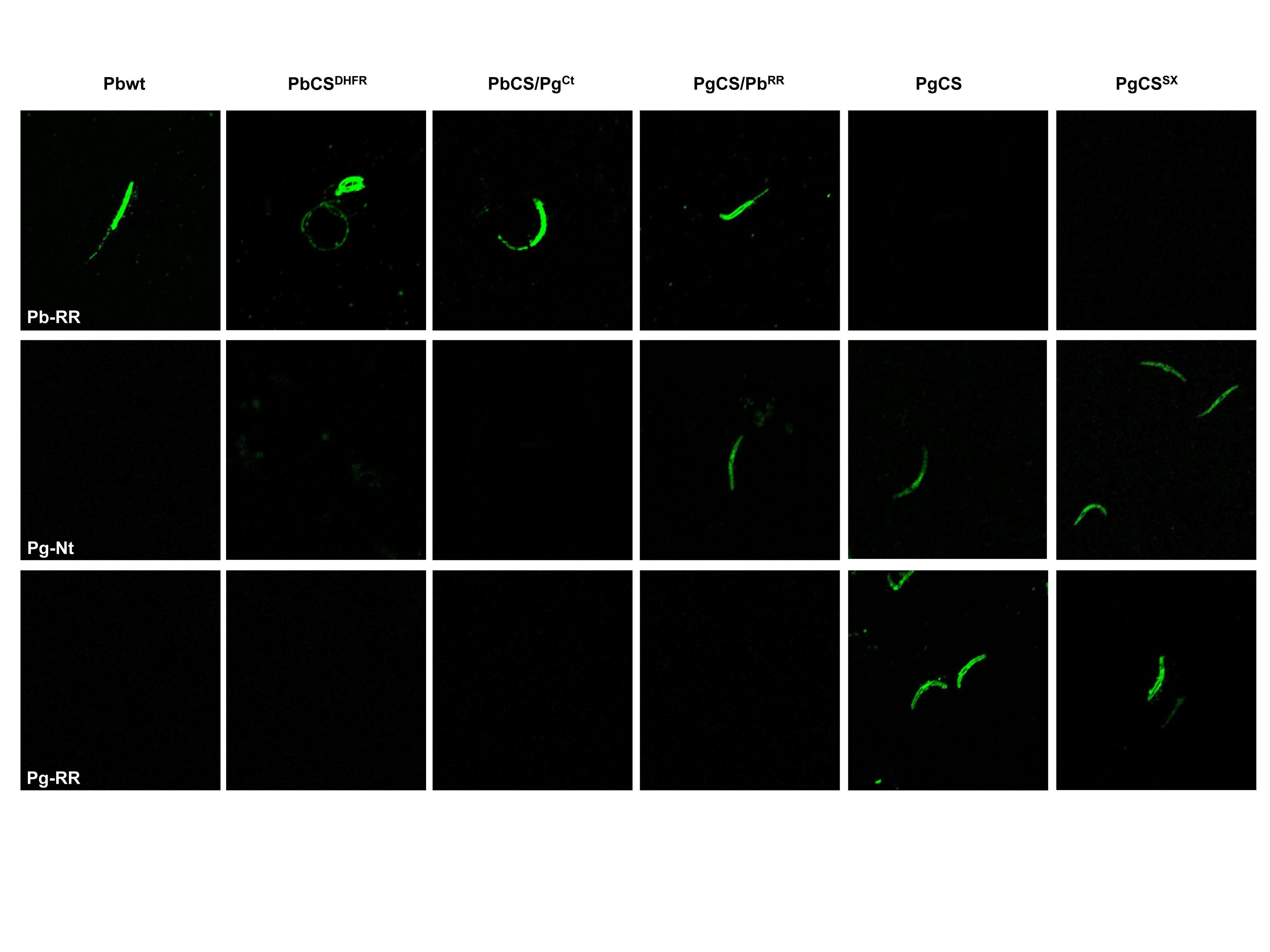

Supplement: Figure S3 — Sporozoite CSP expression and motility. Confocal immunofluorescence microphotographs of P. berghei wt and transgenic midgut sporozoites incubated at 37°C to induce motility and developed with either a monoclonal antibody directed against the PbCSP repeat region (Pb-RR), a serum directed against the PgCSP N-terminal region (Pg-Nt), or a serum directed against the PgCSP repeat region (Pg-RR). Sporozoites shed trails of material recognised by antibody against the PbCSP repeat region. Antibody against the PgCSP N-terminal region (Pg-Nt) revealed mainly an intracellular pattern of expression, and no trails, for the PgCS/PbRR, PgCSSX and PgCS replacement parasites. The antibody against the Pg-RR revealed the presence of clumps of immunoreactive material either outside the parasite body or in proximity to its surface. (TIF) [file pone.0032524.s003.tif]

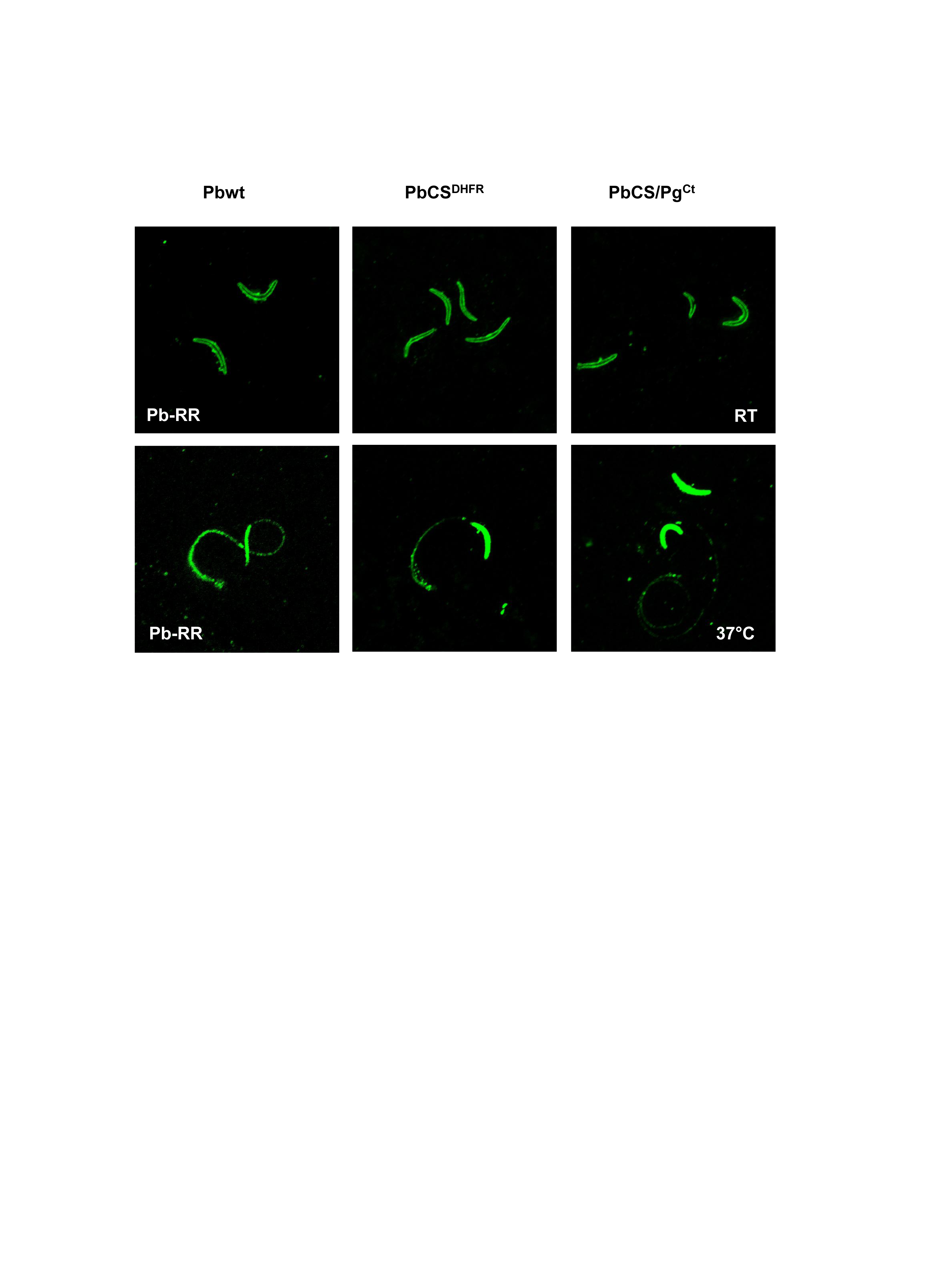

Supplement: Figure S4 — Salivary gland sporozoite CSP expression and motility. Confocal immunofluorescence microphotographs of P. berghei wt and transgenic salivary gland sporozoites incubated at either room temperature (RT) or 37°C and developed with an antibody directed against the PbCSP repeat region (Pb-RR). The antibody revealed surface expression in PbCSDHFR and PbCS/PgCT salivary gland sporozoites, similar to P. berghei wt salivary gland sporozoites. Sporozoites incubated at 37°C shed trails of material recognised by the anti-PbCSP repeat region antibody. (TIF) [file pone.0032524.s004.tif]
